# Increased expression of Myosin X contributes to the metastasis in patients with laryngeal squamous cell carcinoma

**DOI:** 10.1007/s00438-022-01934-x

**Published:** 2022-08-11

**Authors:** Gang Deng, Tie-Jun Fu, Cui-Ping Liu

**Affiliations:** 1 Department of Otolaryngology-Head and Neck Surgery, Wuhan No.1 Hospital, Wuhan, People’s Republic of China; 2 Department of Otolaryngology-Head and Neck Surgery, Shiyan Hospital of Integrated Traditional and Western Medicine, Shiyan, People’s Republic of China; 3 Department of Otolaryngology-Head and Neck Surgery, Second People’s Hospital of Gansu Province, No. 1 He Zheng West Street, Chengguan District, Lanzhou, 730000 Gansu People’s Republic of China

**Keywords:** Myosin X, Laryngeal squamous cell carcinoma, Metastasis, MMP, Epithelial-mesenchymal transition

## Abstract

Laryngeal Squamous Cell Carcinoma (LSCC) is one of the most common malignancy in Head and neck cancer for which the mechanism underlying its metastasis is poorly understood. Myosin X, a molecular motor in cells has been demonstrated to play an important role in cell migration. However, whether Myosin X is involved in the metastasis of LSCC remains unclear. To investigate the expression of Myosin X and its implication in the metastasis of LSCC, we recruited 30 patients with LSCC and 6 patients with vocal cord polyp range from October 2016 to October 2018. Tissue samples were obtained during surgery and the expression of Myosin X, Cortactin, MMP2, MMP9, E-cadherin, and β-catenin in tissue samples were evaluated by RT-PCR, Western blot, immunohistochemistry or ELISA. Patients with LSCC were further followed-up 2 year after surgery for metastasis analysis. We found that the level of Myosin X, Cortactin, MMP2, and MMP9 was much higher in poorly differentiated LSCC compared to that in moderately and highly LSCC, as well as the control tissues. In contrast, the expression of epithelial-mesenchymal transition related marker, E-cadherin, and β-catenin, were much lower in poorly differentiated LSCC tissues compared to that in moderately and highly differentiated LSCC tissues, as well as the control tissues. Moreover, the expression of Myosin X was positively correlated with Cortactin, MMP2, and MMP9 levels. Increased expression of Myosin X in LSCC tissues was related to higher risk of metastasis. In conclusion, our findings showed that. Myosin X augments the expression of Cortactin, MMP2 and MMP9, which could upregulate the cell migration and the matrix degradation, and consequently reduce the expression of E-cadherin and β-catenin, thereby activating epithelial-mesenchymal transformation and promoting the metastasis of LSCC. Targeting Myosin X may have potential therapeutic effect in the metastasis of LSCC.

## Introduction

Laryngeal squamous cell carcinoma (LSCC) is one of the most common malignancy in head and neck cancer (Genden et al. 2007). Despite the availability of combined modality treatment such as surgery, chemotherapy, radiotherapy, and molecular targeted therapies in the treatment of LSCC patients, LSCC is reported to have a poor prognosis with a low survival rate (Olsen 2010; Rudolph et al. 2011). One of the key factors is the metastasis of LSCC in these patients. The metastasis of cancer is a complex process involving multiple factors. Generally, carcinoma cells growing in the primary site can degrade the extracellular matrix, invade across the basement membrane, and subsequently enter the lymphatic lumen or capillary walls. Circulating carcinoma cells then adhere to the distal vascular endothelial cells and infiltrate into remote tissues. Metastatic tumors are formed through the malignant proliferation of carcinoma cells (Hristov and Bajaj 2008). Therefore, elucidating the factors leading to the metastasis of LSCC remains a research focus.

Cell migration is a highly integrated process that essential for tumor invasion and metastasis. Filopodia on the leading edge of the tumor cell may play an important role in the initial stage of cell invasion. Myosin X is an important molecular motor in cells, which is mainly expressed in filopodia at the protuberant part of cell membrane, and, therefore, may play an important role in cell migration. Recent studies have shown that Myosin X bind to Cortactin, an actin-related protein complex, and participate in cell migration, thus playing an important role in the occurrence, invasion and metastasis of tumors (MacGrath and Koleske 2012). In addition, Myosin X can also activate molecular signaling, leading to the degradation of extracellular matrix and the mesenchymal transdifferentiation to epithelial cells (Sun et al. 2019). Degradation of extracellular matrix (ECM) and basement membrane by tumor cells is a key step in tumor invasion and metastasis. Emerging evidence have shown that matrix metalloproteinases (MMP) can degrade matrix and basement membrane, and, therefore, promoting the infiltration and metastasis of tumor cells (Gabison et al. 2005; Scheau et al. 2019). Increased Myosin X expression has been shown in breast cancer cells and is closely related to the lymph node metastasis and the invasiveness of cancer (Gabison et al. 2005). However, the expression of Myosin X in LSCC and whether Myosin X is associated with the metastasis of LSCC remains unclear. The present study was undertaken to explore (Genden et al. 2007) the expression of Myosin X in LSCC; (Olsen 2010) Whether Myosin X are implicated in the metastasis of LSCC.

## Method

### Subjects

This study was approved by the Ethics Committee of Wuhan No.1 Hospital and conducted with written informed consent from every patient. A total of 30 patients with LSCC admitted to Wuhan NO.1 hospital from October 2016 to October 2018 were enrolled. Inclusion criteria were (1) The clinical manifestations and pathological findings were in accordance with the diagnosis of LSCC; (2) All patients with LSCC were admitted to Wuhan NO.1 hospital without any preoperative radiotherapy or chemotherapy; (3) All patients have complete medical records. Patients who have absolutely contraindication of operation were excluded. Thirty patients with LSCC (including 18 male and 12 female, mean age 34.8 ± 7.6 years) were further classified into poorly differentiated group, moderately differentiated group, and well differentiated group based on the histology sections evaluated by two independent physicians who were blind to the clinical data. Subjects undergoing polypectomy because of vocal cord polyp and without other laryngeal diseases were enrolled as control subjects. Not all samples were included in every experimental protocol because of limited quantity.

Patients with LSCC were followed and examined monthly for the first 6 months and then every 2 months for the second half of the year, and every 3 months for the 2nd year after surgery. In the follow-up assessments, office endoscopic examinations were done at each visit, and neck and chest CTs were obtained annually.

### Histology and immunohistochemistry

Paraffin sections (4 μm) were prepared from paraffin-embedded human tissue samples. After deparaffinization and rehydration, sections were stained with hematoxylin and eosin to determine the histology and differentiation of LSCC. Sections were further subjected to heat-induced epitope retrieval using citric acid repair solution (Guge Biotechnology, Wuhan, China). The 3% hydrogen peroxidase (Boster Biotechnology, Wuhan, China) was used for endogenous peroxidase inhibition and 5% bovine serum albumin (Guge Biotechnology) was used to block nonspecific binding. Sections were stained with primary antibodies against E-cadherin (1:100, Abcam, Cambridge, UK) and β-catenin (1:100, Abcam) overnight at 4 °C. Antigens were detected using the streptavidin–peroxidase complex method with a histostain-plus kit (Boster Biotechnology) according to the manufacturer’s instructions. Color development was achieved with 3ʹ, 3ʹ-diaminobenzidine, which rendered positive cells brown.

### Quantitative RT-PCR

Total RNA was extracted from tissue samples using TRIzol reagent (Ambion, Shanghai, China) according to the manufacturer’s instructions. A total of 500 ng RNA was reverse-transcribed to cDNA and cDNA equivalent to 25 ng of total RNA was used for PCR assay. Quantitative PCR was performed using the SYBR PCR kit (KAPA Biosystems, Shanghai, China) with specific primers on CFX-Connect 96 (Bio-Rad, Hercules, Calif., USA). The sequences were as follows: MMP2: forward primer, 5ʹ-TACAGGATCATTGGCTACACACC-3ʹ, reverse primer, 5ʹ-GGTCACATCGCTCCAGACT-3ʹ; MMP9: forward primer, 5ʹ-TGTACCGCTATGGTTACACTCG-3ʹ, reverse primer, 5ʹ-GGCAGGGACAGTTGCTTCT-3ʹ; Myosin X: forward primer, 5ʹ-GAACCCCTACCAGCCCATC-3ʹ, reverse primer, 5ʹ-GTTTTACCTGCCCCACTTTCA-3ʹ; Cortactin: forward primer, 5ʹ-GTGGTTTTGGCGGCAAGTATG-3ʹ, reverse primer, 5ʹ-CTCTCTGTGACTCGTGCTTCT-3ʹ; Glyceraldehyde-3-phosphate dehydrogenase (GAPDH): forward primer, 5ʹ-GGAGCGAGATCCCTCCAAA-3ʹ, reverse primer, 5ʹ-GGAGCGAGATCCCTCCAAAAT-3ʹ. Amplification was as follows: 95 °C for 2 min, followed by 40 cycles of 95 °C for 10 s, specific annealing temperature for 10 s, and 72 °C for 15 s. After PCR, a melting curve was constructed by increasing the temperature from 65 to 95 °C with a temperature transition rate of 0.1 °C/s. Relative gene expression was calculated using the 2(-Delta Delta CT) method. GAPDH was used as a housekeeping gene for normalization of gene expression.

### Tissue homogenate preparation and ELISA

Tissue samples were weighed and 1 mL of 0.9% sodium chloride solution supplemented with 10 μL of 100 mM phenylmethylsulfonyl fluoride was added per 100 mg tissue. Tissues were then homogenized on ice and centrifuged at 12,000 rpm for 15 min at 4 °C. Supernatants were harvested and stored at − 80 °C for future studies. MMP2 and MMP9 levels in supernatants were measured using commercial kits (Bioswamp, Wuhan, China) according to the manufacturer’s instructions.

### Western blot analysis

Total protein was extracted from tissues and measured with a BCA protein assay kit (Bioswamp, Wuhan, China). Samples containing 20 μg of proteins were separated by 12% sodium dodecyl sulfate–polyacrylamide gel electrophoresis under reducing conditions and transferred to polyvinyl difluoride membranes (Guge Biotechnology, Wuhan, China). After blocking with 5% fat-free skim milk for 1 h at room temperature, the membranes were incubated overnight with primary antibodies against Myosin X (1:100, Abcam, Cambridge, UK) and Cortactin (1:100, Abcam). After washing three times in TBS-T buffer, the membranes were incubated with horseradish peroxidase-conjugated secondary antibody for 1 h at room temperature. The membranes were then processed using an ECL chemiluminescence reaction kit and followed by exposure on chemiluminescent film to visualize the proteins. GAPDH quantification was used as an internal standard to correct for variations in total protein loading. Densitometric analysis of the blots was performed using the software TANON GIS.

### Cell culture and transfection

Human LSCC cell lines (AMC-HN-8) were purchased from the Cell Biology Institute of Shanghai, Chinese Academy of Science (Shanghai, china). AMC-HN-8 cells were maintained in Dulbecco's modified Eagle’s medium (DMEM, Gibco, Grand Island, NY, USA) supplemented with 10% fetal bovine serum (FBS, Gibco) and incubated at 37 °C in 5% CO_2_ humidified incubator. Cells were transfected with a synthetic siRNA targeting human Myosin X (5ʹ-AAGTGCGAACGGCAAAAGAGA-3ʹ) or a control siRNA for 48 h using Lipofectamine 3000.

### Statistical analysis

Expression data are presented in dot plots unless specifically indicated. The Kruskal–Wallis *H* test was used to assess significant intergroup variability and the Mann–Whitney *U* two-tailed test was used for between-group comparison. Correlations were performed using Spearman’s correlation. *P* < 0.05 was considered significant.

## Results

### The histology of tissues from patients with LSCC and control subjects

**Fig. 1:**
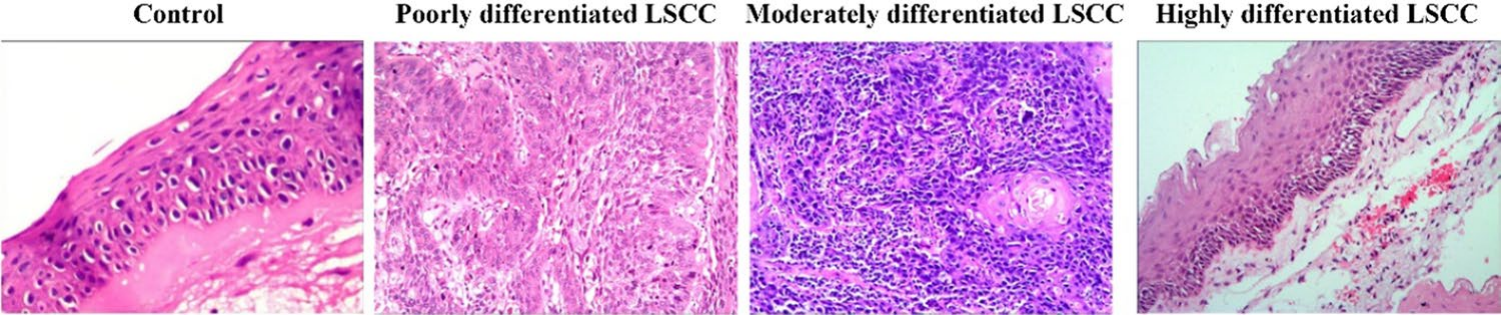
The histology of tissues from patients with LSCC and control subjects. Representative photomicrographs showing the histology of tissues from patients with LSCC and control subjects (original magnification × 400). LSCC, laryngeal squamous cell carcinoma

We first compare the histology of LSCC and control tissues. We classified tissues from LSCC into poorly differentiated group, moderately differentiated group, and well differentiated group and found that the histology of tissue from LSCC is quite distinct from control tissues (Fig. 1).

### Increased expression of Myosin X in LSCC tissues

**Fig. 2:**
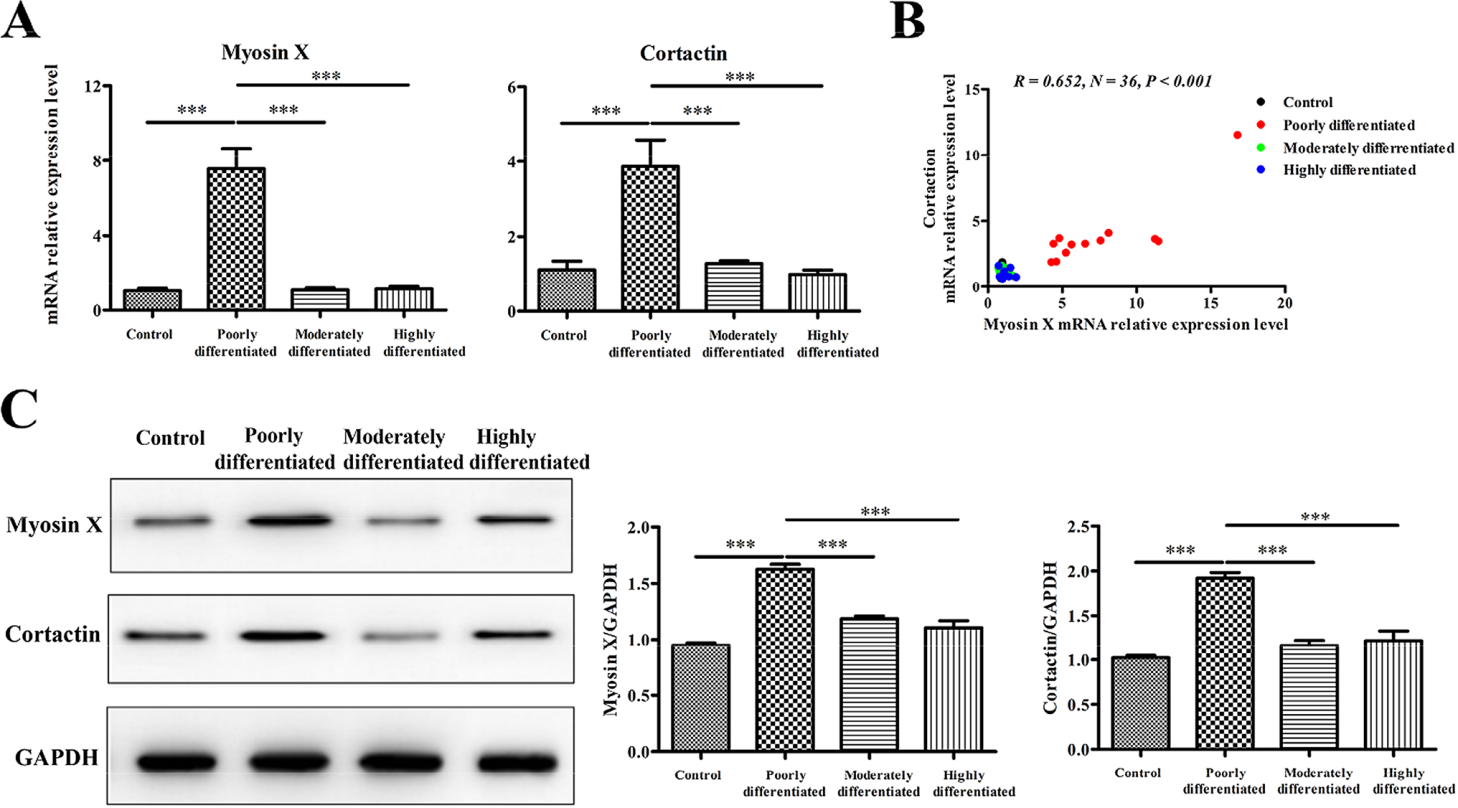
Increased Myosin X and Cortactin expression in tissues from LSCC. **A** The mRNA expression levels of Myosin X and Cortactin in tissue samples in different study groups as detected by quantitative RT-PCR. Control, *n* = 6; poorly differentiated, *n* = 12; moderately differentiated, *n* = 9; highly differentiated, *n* = 9; **B** Myosin X mRNA expression positively correlated with Cortactin mRNA in tissues in patients with LSCC; **C** the protein levels of Myosin X and Cortactin in tissue samples in different study groups as detected by western blotting. Control, *n* = 5; poorly differentiated, *n* = 5; moderately differentiated, *n* = 5; highly differentiated, *n* = 5. ^***^P < 0.001

We found that in comparison with those in control tissues, the mRNA expression level of Myosin X was significantly upregulated in poorly differentiated LSCC tissues compared that in moderately differentiated LSCC tissues, well differentiated LSCC tissues and control tissues. Myosin X can bind to Cortactin to exert its function in cell migration, we further found that the mRNA expression level of Cortactin was significantly upregulated in poorly differentiated LSCC tissues compared that in moderately differentiated LSCC tissues, well differentiated LSCC tissues and control tissues (Fig. 2A). Moreover, the mRNA level of Cortactin was positively correlated with the transcription of Myosin X (Fig. 2B). The upregulation of Myosin X and Cortactin in LSCC tissues were further confirmed at the protein level by western blot analysis (Fig. 2C).

### Increased expression of MMP2 and MMP9 in LSCC tissues

**Fig. 3:**
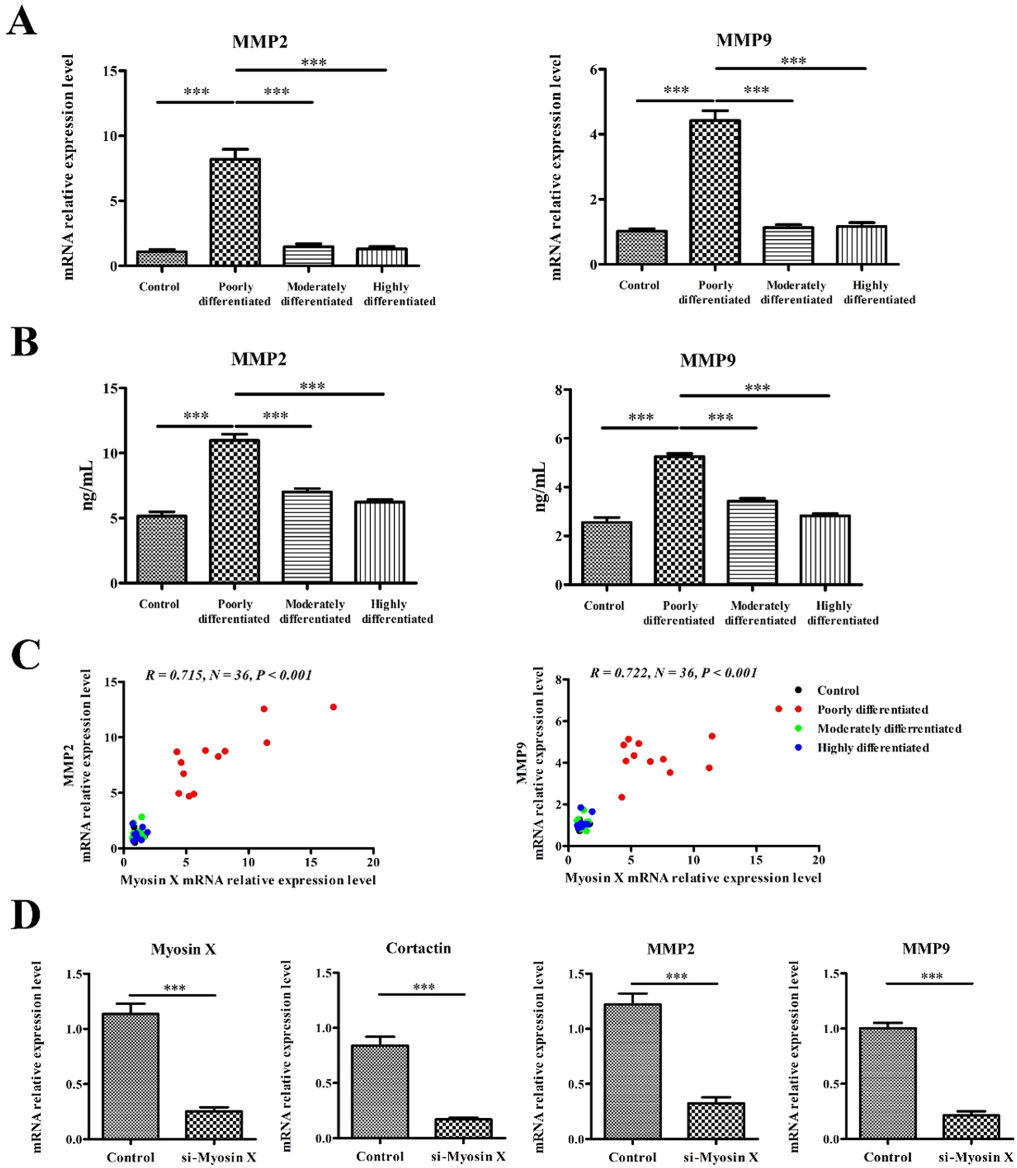
Increased MMP2 and MMP9 expression in tissues from LSCC. **A** The mRNA expression levels of MMP2 and MMP9 in tissue samples in different study groups as detected by quantitative RT-PCR. Control, *n* = 6; poorly differentiated, *n* = 12; moderately differentiated, *n* = 9; highly differentiated, *n* = 9; **B** the protein levels of MMP2 and MMP9 in tissue samples in different study groups as detected by ELISA. Control, *n* = 6; poorly differentiated, *n* = 12; moderately differentiated, *n* = 9; highly differentiated, *n* = 9; **C** Myosin X mRNA expression positively correlated with MMP2 and MMP9 mRNA in tissues in patients with LSCC. ^***^P < 0.001. **D** AMC-HN-8 cells were transfected with siRNA targeting Myosin X or control siRNA for 48 h. Cells or cell culture supernatants were subjected to RT-PCR assay (*n* = 5). ^***^P < 0.001

MMP2 and MMP9 levels were associated with the metastasis of LSCC. We further found that the mRNA expression of MMP2 and MMP9 level was upregulated in poorly differentiated LSCC tissues compared to that in moderately differentiated LSCC tissues, well differentiated LSCC tissues and control tissues (Fig. 3A). This finding was further confirmed at protein level by ELISA (Fig. 3B). Correlative analysis revealed that mRNA expression of Myosin X level positively correlated with MMP2 and MMP9 levels (Fig. 3C), suggesting that Myosin X may have effect on the regulation of MMP in LSCC. To test this possibility, we treated AMC-HN-8, a LSCC cell line with siRNA targeting Myosin X. Our results revealed that the expression of Cortactin, MMP2 and MMP9 were significantly downregulated by si-Myosin X treatment, further supporting this notion (Fig. 3D).

### Decreased expression of E-cadherin and β-catenin in LSCC tissues

**Fig. 4:**
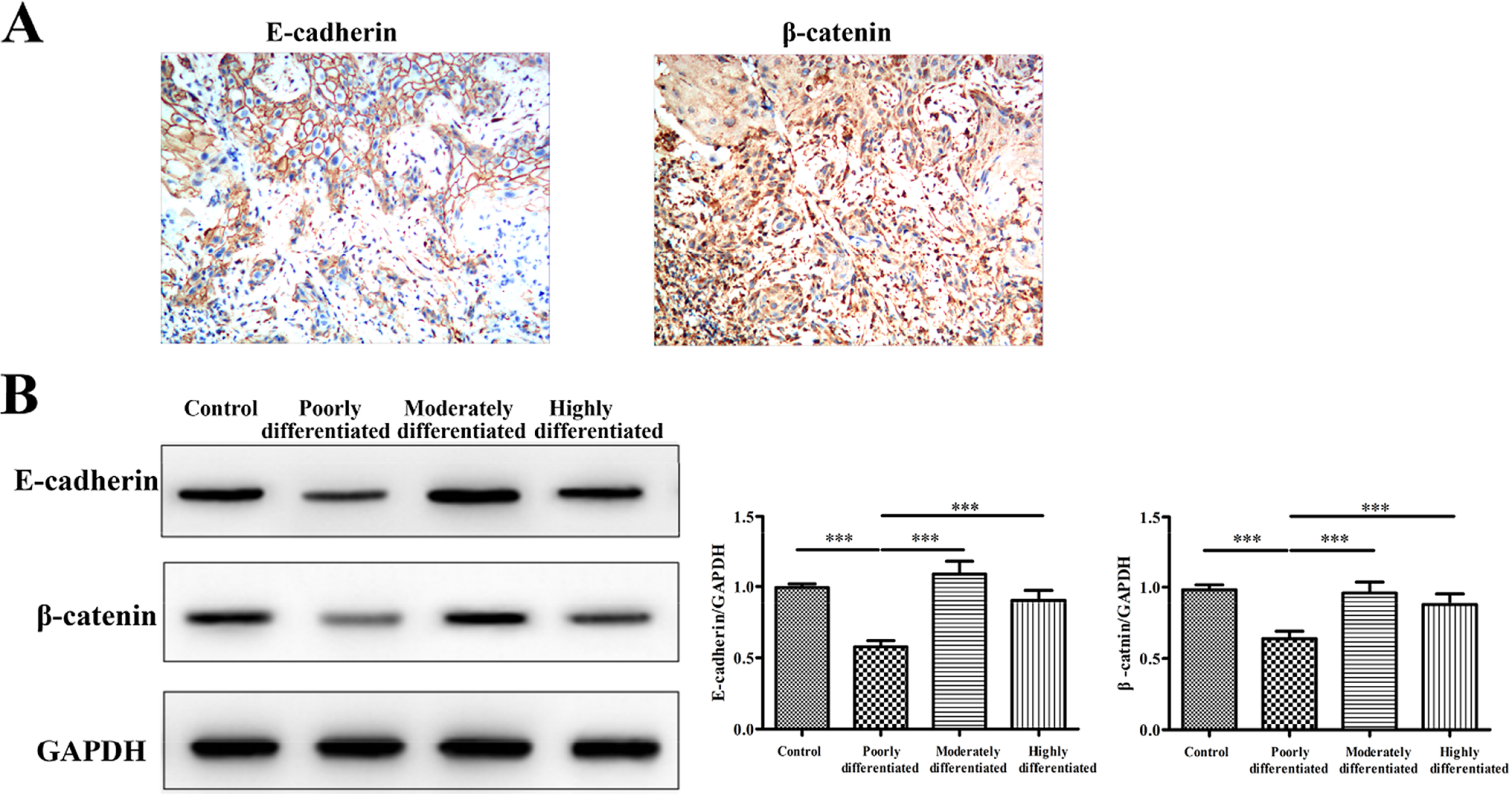
The expression of E-cadherin and β-catenin in tissues from LSCC. **A** The immunoreactivity of E-cadherin (left panel) and β-catenin (right panel) in LSCC tissues. The representative photomicrographs of a highly differentiated LSCC sample are shown (original magnification × 400); **B** the protein levels of E-cadherin and β-catenin in tissue samples in different study groups as detected by western blotting. ^***^P < 0.001

We next explore the expression of epithelial-mesenchymal transformation (EMT) related marker, E-cadherin and β-catenin in LSCC. We detected the expression of E-cadherin and β-catenin in LSCC tissue by immunohistochemistry (Fig. 4A). We further found that E-cadherin and β-catenin levels were decreased in poorly differentiated LSCC tissues compared to that in moderately differentiated LSCC tissues, well differentiated LSCC tissues and control tissues by western blot analysis (Fig. 4B).

### Elevated expression of Myosin X correlates with lymph node metastasis

**Fig. 5:**
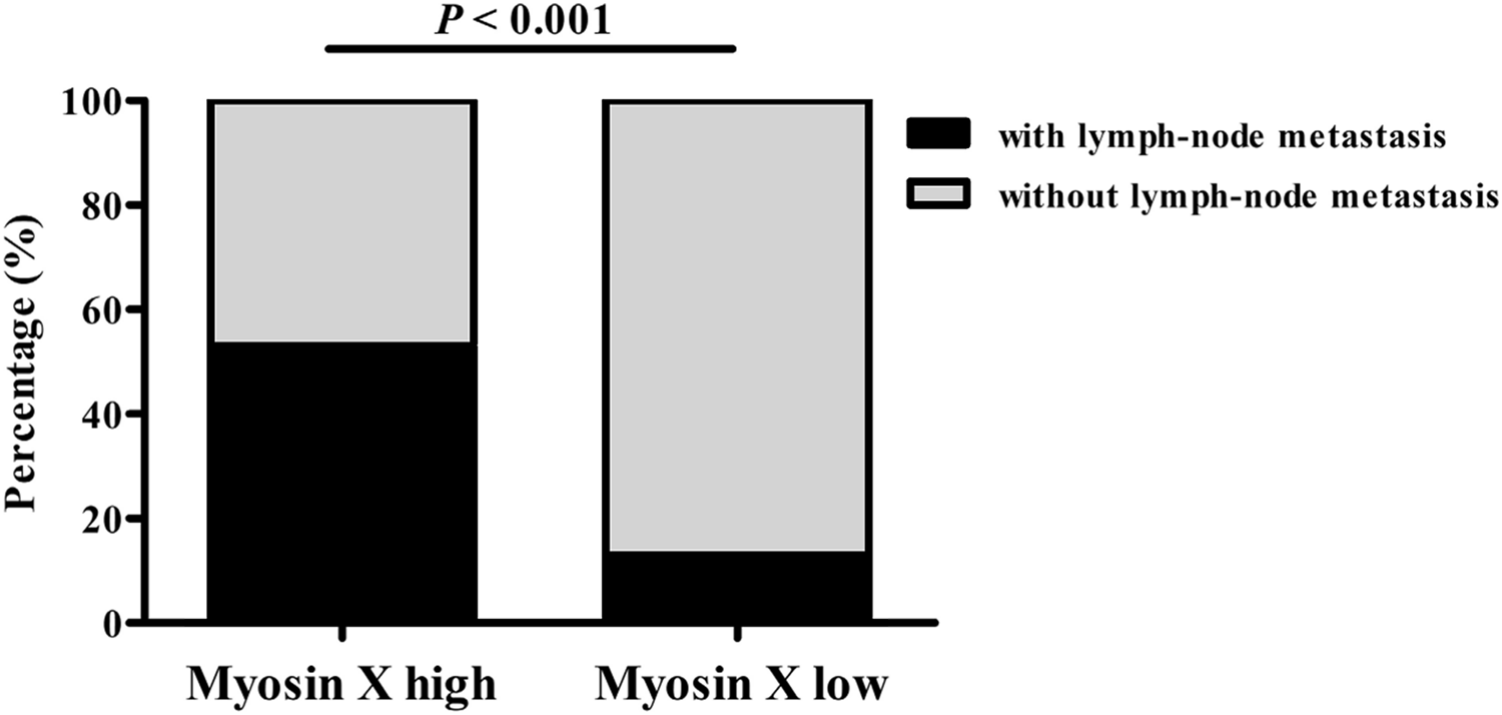
Elevated expression of Myosin X correlates with lymph node metastasis. The correlation between Myosin X expression and lymph node metastasis by a 2-year follow-up

To determine the clinical significance and underlying role of Myosin X in LSCC, we made a 2-year follow-up and analyzed the correlations between the expression of Myosin X and lymph node metastasis. None of LSCC patients were died at the 2-year follow-up. We found that increased Myosin X expression correlated with lymph node metastasis in LSCC (*P* < 0.001, Fig. 5).

## Discussion

LSCC is the most common pathological phenotype of laryngeal carcinoma. LSCC, especially poorly differentiated LSCC, is associated with high degree of malignancy, showing high potential of metastasis, leading to the low postoperative survival rate. Lymph node metastasis has been previously shown closely related to the prognosis of LSCC (Bayir et al. 2021). Therefore, understanding the pathogenesis of LSCC, especially the molecular mechanism contributing to the metastasis, is beneficial to the early diagnosis and treatment of this disease (Olsen 2010; Rudolph et al. 2011). In this study, we investigated the expression of Myosin X in LSCC tissues. Our results demonstrated that Myosin X expression was elevated in poorly differentiated LSCC, and the level of Myosin X was positively correlated with the expression of Cortactin, as well as MMP2 and MMP9 levels, suggesting that Myosin X may contribute to the metastasis of LSCC by regulating cell migration and MMP expression.

Cell adhesion mainly occurs in the forms of cell–extracellular matrix and cell–cell adhesion, and a change of cell adhesion is necessary for tumor cells to obtain the ability to metastasize. EMT is a key factor responsible for the tumor metastasis and distal lesion formation. E-cadherin is one of the main adhesion molecules, and its expression has been reported to be significantly decreased during tumor EMT (Pastushenko and Blanpain 2019). Mechanically, the reduction of E-cadherin lead to the loss of cell adhesion and the loss of interaction and polarity between tumor cells, therefore, enhancing the ability of tumor cell invasion and migration (Pastushenko and Blanpain 2019). EMT is the main cause of metastasis and invasion of many malignant tumors, which has been found in gastric cancer, lung cancer, and colon cancer (Pastushenko and Blanpain 2019), although the underlying mechanisms contributing to EMT activation is different depending on the different context of tumor tissues (Pastushenko and Blanpain 2019). EMT may play a role in the metastasis of LSCC as well. Therefore, exploring the factors regulating EMT in LSCC is expected to provide a new strategy for the diagnosis, treatment and prognosis of LSCC.

To explore the role of Myosin X in the metastasis of laryngeal cancer, we examine both the mRNA and protein level of Myosin X in LSCC tissues. We found that the expression level of Myosin X in poorly differentiated LSCC tissues was significantly upregulated compared with that in moderately differentiated LSCC tissues, highly differentiated LSCC tissues, and control tissues, suggesting that Myosin X may be associated with the metastasis of LSCC. Interestingly, we found that the expression level of E-cadherin in poorly differentiated laryngeal cancer tissues was significantly lower than that in moderately differentiated laryngeal cancer tissues and highly differentiated laryngeal cancer tissues, implicating that EMT plays an important role in the metastasis of LSCC. Previous studies have shown that MMP can destroy E-cadherin and thus promoting EMT (Boukhedouni et al. 2020). MMP2 and MMP9 are members of MMPs family and play a pivotal role in cell migration during physiological and pathological processes (Wang et al. 2006). We further found that the expression of MMP2 and MMP9 in poorly differentiated LSCC was significantly higher than that in moderately differentiated LSCC, highly differentiated LSCC and controls, therefore, the decrease of E-cadherin in poorly differentiated LSCC may due to the increased expression of MMP2 and MMP9. Furthermore, we found that Myosin X was positively correlated with the expression levels of MMP2 and MMP9, suggesting that Myosin X may be involved in the EMT of LSCC through the regulation of MMP, which may contribute to the metastasis of LSCC. This notion was further supported by our 2-year follow-up and we found that patients with higher Myosin X expression had increased risk of lymph node metastasis in patients with LSCC.

Our study has several limitations. First, it has been shown that supraglottic, glottic, and subglottic laryngeal cancers may have different risks of lymph node metastases. However, the sample size of the present study does not allow adequate stratification of patients by subsite, which deserves further study. Second, the association between the expression of Myosin X and Cortactin, MMP2, and MMP9 in vivo in patients with LSCC should further be confirmed in vitro study.

Nevertheless, in summary, our study is the first to explore the expression of Myosin X in LSCC patients and implicate that Myosin X may promote the expression of Cortactin, MMP2 and MMP9, downregulate the expression of E-cadherin, activate the EMT of LSCC, and thus promoting the formation of metastatic lesions. Targeting Myosin X may, therefore, have potential therapeutic effect in the metastasis of LSCC.
